# Ultrasonic acoustic levitation for fast frame rate X-ray protein crystallography at room temperature

**DOI:** 10.1038/srep25558

**Published:** 2016-05-06

**Authors:** Soichiro Tsujino, Takashi Tomizaki

**Affiliations:** 1 Department of Synchrotron Radiation and Nanotechnology, Paul Scherrer Institute, 5232 Villigen-PSI, Switzerland

## Abstract

Increasing the data acquisition rate of X-ray diffraction images for macromolecular crystals at room temperature at synchrotrons has the potential to significantly accelerate both structural analysis of biomolecules and structure-based drug developments. Using lysozyme model crystals, we demonstrated the rapid acquisition of X-ray diffraction datasets by combining a high frame rate pixel array detector with ultrasonic acoustic levitation of protein crystals in liquid droplets. The rapid spinning of the crystal within a levitating droplet ensured an efficient sampling of the reciprocal space. The datasets were processed with a program suite developed for serial femtosecond crystallography (SFX). The structure, which was solved by molecular replacement, was found to be identical to the structure obtained by the conventional oscillation method for up to a 1.8-Å resolution limit. In particular, the absence of protein crystal damage resulting from the acoustic levitation was carefully established. These results represent a key step towards a fully automated sample handling and measurement pipeline, which has promising prospects for a high acquisition rate and high sample efficiency for room temperature X-ray crystallography.

X-ray diffraction is one of the most-established methods for the structural analysis of macromolecules. It is essential for the determination of the atomic configuration of biological crystalline specimens and for the investigation and confirmation of the 3-dimensional coordination of synthetic molecules. By combining synchrotron light sources with 2-dimensional high frame rate pixel detectors such as the PILATUS series^1^, the collection time for a complete dataset has been reduced to a few minutes. Acquisition rates that are now routinely above 10 Hz enable the continuous rotation of the protein crystal maintained at cryogenic temperatures. Such an X-ray diffraction experiment setup, which is common to all major synchrotron beamlines, has accelerated the solution of important protein molecular structures over the past decade^2^. It has been proposed that an even more rapid data acquisition rate of 100–1000 Hz or higher may lead to the minimal damage of fragile protein crystals by outrunning the diffusion of radicals generated by X-ray absorption^3^, thus drastically improving the quality of a diffraction dataset at room temperature. A more rapid data acquisition rate^4^ would also make it possible to perform a range of experiments, such as *in-situ* structural dynamics studies and time-resolved experiments, that can be conducted only at room temperature, at which the protein crystals is closer to physiological conditions. Room temperature experiments will not only enable the rapid screening of crystals but also facilitate the investigation of the conformational diversity of biomolecules that are difficult to study with cryo-cooled specimens^5^,^6^. The method explored in this study may potentially make such room temperature experiments realistic: a combination of a next-generation pixel detector with a frame rate of 133 Hz coupled with the X-ray diffraction of spinning protein crystals in droplets that are levitated in air by ultrasonic acoustic pressure^7^. Acoustic waves have previously been used to levitate droplets and small particles^8^,^9^,^10^, metal particles^11^, living insects^12^, and fish embryos^13^, for manipulation and transportation and as a means to conduct various physical^14^,^15^,^16^, chemical^17^,^18^,^19^,^20^,^21^ and biochemical^22^,^23^,^24^ analyses in a container-less environment. Acoustic pressure has also been used to load crystals on a micro mesh^25^ and a solid conveyor belt^26^ for X-ray crystallography or to set up crystallization trials^27^,^28^, but this is the first time that acoustic levitation has been used to solve the structure of protein crystal samples from the acquired X-ray diffraction images.

When a crystal is inside a levitating liquid droplet, the internal circulation of the droplet caused by acoustic streaming^14^,^29^,^30^,^31^,^32^ induces the rapid spinning and orbiting of the crystal. Consequently, careful alignment of the X-ray beam to the crystal allows the acquisition of a dataset that encompasses various crystal orientations in a short time. Although conventional oscillation methods that use the serial variation of the crystal orientation are advantageous for rapid data processing, the recently developed data processing methods for serial femtosecond crystallography (SFX) can analyse a dataset that contains random crystal orientations for indexing and solving the protein structure, as long as the entire variation of the crystal orientation in the dataset leads to complete diffraction spots as shown below. The addition of a sample injection mechanism^25^ to the acoustic levitator described in this study might also be used to actualize fully automated X-ray diffraction experiments with a high data acquisition rate and efficiency, thereby accelerating both the structural analysis of biomolecules and structure-based drug development^33^,^34^,^35^. However, it is crucial to ensure that acoustic levitation is compatible with fragile macromolecular crystals.

In this study, we constructed an acoustic levitator and performed single crystal X-ray diffraction experiments on beamline X06SA at the Swiss Light Source (SLS) at the Paul Scherrer Institut (PSI) using a fast frame rate pixel detector. From the acquired diffraction images, we were able to determine a refined protein crystal structure at high resolution. Our results show that the method is promising as a liquid-phase sample delivery method that does not cause physical damage to the crystals for end users in protein crystallography.

## Results

### Ultrasonic acoustic levitator

**Figure 1:**
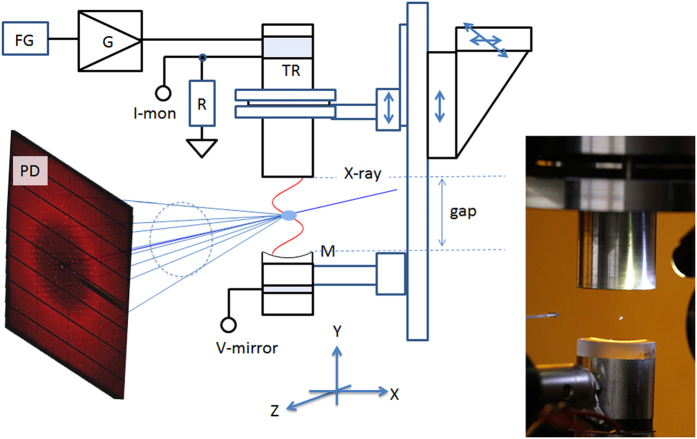
X-ray diffraction of protein crystals in levitated liquid droplet. In the schematic below, FG: function generator, G: power amplifier, TR: ultrasonic transducer, M: ultrasonic mirror reflector, PD: X-ray pixel detector. The oscillation amplitude of TR was feedback controlled with the driving current, I-mon. TR and M form an acoustic cavity. With the V-mirror signal, the cavity gap was set to control the cavity acoustic pressure for stable droplet levitation. The X-ray beam irradiates the levitated droplet along the z-axis. The picture on the right shows a 4 μ l droplet containing single protein crystal when it was stably levitated at the second node of the cavity.

The acoustic levitator, illustrated in Fig. 1, is an acoustic cavity that consists of an ultrasonic transducer with a resonance frequency of ~38 kHz (a wavelength of ~9 mm in ambient air at 23 °C) and a concave mirror reflector. When the cavity gap between the transducer and the reflector is an integer multiple of the half wavelength of the ultrasonic acoustic radiation, liquid droplets can be trapped at pressure nodes. Because the holding force from the acoustic trapping is stronger than gravity, the droplet levitates in the air. By adjusting the gap to the third resonance and loading liquid droplets in the middle node, diffraction angles up to ~41° are accessible. For diffraction experiments, we set the peak cavity acoustic pressure to 2.1 ±  0.1 kPa (rms) and loaded a 4-μ l-droplet of crystallization liquid containing single crystals. At this pressure, the droplet was levitated and assumed an ellipsoidal shape with a positional stability of a few tens of micrometres over a time scale of a few minutes, which was sufficient for the X-ray diffraction experiments. The evaporation of the crystallization buffer and the associated change in the concentration of the solution during such a time interval are minimal, as is the temperature increase^15^. The estimated volume reduction in 3 min is at most, ~0.05 μ l. The estimated pressure inside a levitated droplet is several orders of magnitude less than the peak cavity pressure because the droplets are levitated at the pressure node. Additionally, because the acoustic impedance of the liquid is 3 orders of magnitude higher than that of the air, the cavity pressure transmitted into the droplet is reduced by the same amount. The most important factor for an X-ray diffraction experiment is the internal circulation of the levitated droplet induced by the acoustic streaming of the surrounding air^14^,^29^,^30^,^31^,^32^ and the resulting rapid spinning of the crystal orientation inside the droplet. Because of this spinning, additional instrumentation for sample oscillation and rotation, which is crucial for conventional methods, are not required.

### X-ray diffraction experiment

**Figure 2:**
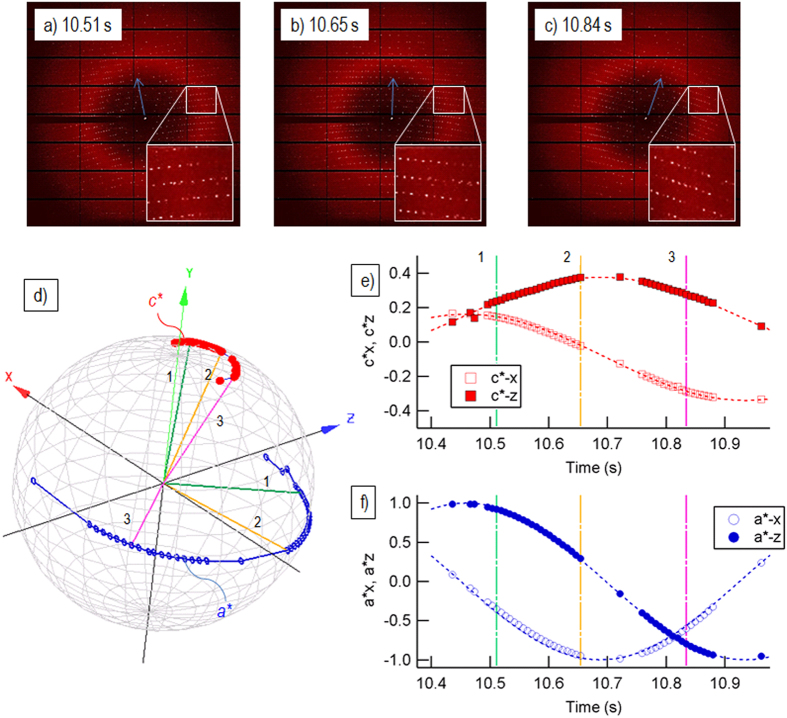
Snapshots of the X-ray diffraction images from one of the datasets. (**a–c**) were acquired, respectively, at 10.51 s, 10.65 s, and 10.84 s after the start of data acquisition. From the indexed diffraction spots, we evaluated the time evolution of the crystal orientation. The directions of a* and c* are indicated on the Gauss plot (**d**), together with the evolution of a* and c* for the time between 10.4 s and 10.9 s: the crystal spins with c* direction as the spin axis about ~1 rotation per second, corresponding to 1–2° per frame. In this example, c* was nearly along the y-direction. (**e,f**) respectively, show the x- and z-component of the unit vectors parallel to a* and c* within this time interval. The time marked as 1, 2, and 3 in (**d–f**), respectively corresponds to the acquisition time of the images (**a–c**).

The levitator was installed on beamline X06SA at the SLS/PSI (see Fig. 1). We enclosed the levitator in a box to avoid drift of the droplet position due to the ambient air flow. A 100-μ m-thick kapton film was used as the window or the transmission of the X-ray diffraction. We used an EIGER X 16 M detector^4^ to acquire the diffraction images, which had a data acquisition rate of 133 Hz and a short readout dead time of 3 μ s (Dectris, Baden, Switzerland). With this detector, a dataset of 3,600 diffraction images per run could be collected in a total duration of ~30 s. By aligning the X-ray beam path with the crystal by adjusting the levitation position, we were able to collect such a dataset with a hit rate of nearly 100%; i.e. nearly all of the frames recorded a crystalline diffraction pattern. We repeated this 2–4 times for each droplet and collected a total of 36,000 diffraction images (10 EIGER 16 M datasets) from 8 crystal-containing droplets. Diffraction images were recorded with a maximum resolution of 1.8 Å in this set of experiments using a detector distance of 300 mm. Figure 2 shows snapshots of the diffraction images separated by 50–100 ms taken from a dataset (also see supplemental movie 1). A variation of the crystal orientation (c^*^ direction) is visible from the movement of the diffraction spots (see below). Figure 2(d–f) depict the actual orbit of the crystal spinning directions between 10.4 s and 10.9 s, visualized as the intersection of the two lattice vectors a* and c* with a unit radius sphere. The large sinusoidal movement of a* [Fig. 2(f)] comparing to the movement of c* [Fig. 2(e)] shows that the crystal spins along c*. At the same time, the spinning axis (c*) makes a small precession, which is beneficial for uniformly sampling the reciprocal lattice points.

### Data processing and crystal structure

**Figure 3:**
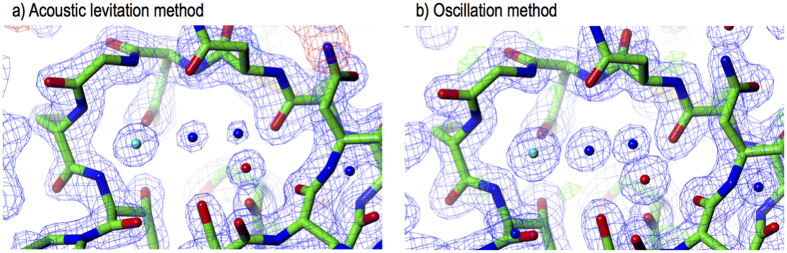
Comparison of the electron density solved and refined from the acoustic levitation method (**a**) and from the conventional oscillation method (**b**). The figures depict a part of 2Fo-Fc electron density maps by CCP4MG^47^. The electron density maps were calculated at 1.5 σ . The contour level of the Fo-Fc map is at ±3 σ . Sodium (red) and Chloride (cyan) atoms and water molecules (blue) are also shown.

**Figure 4:**
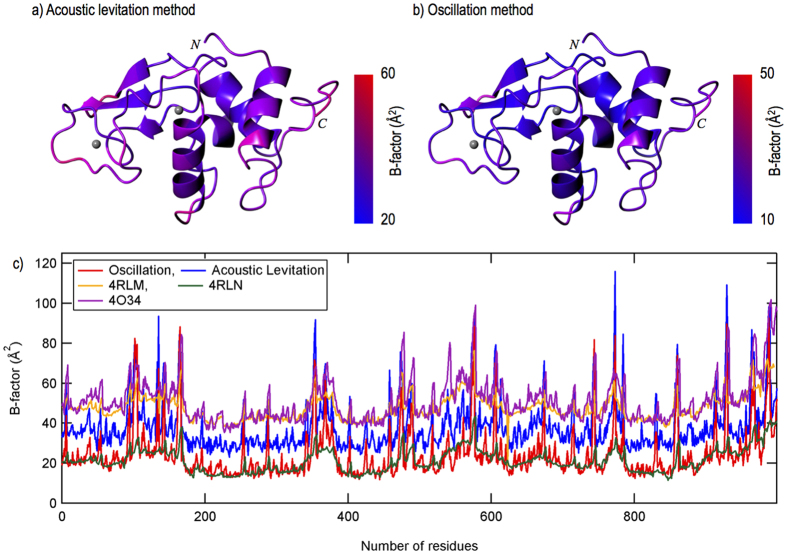
(**a,b**) Show the room temperature lysozyme structures that were solved and refined using the dataset obtained from the acoustic levitation method (**a**) and from the conventional oscillation method (**b**). (**c**) Comparisons of constituent atoms of the lysozyme structures processed with different data processing programs. *XDS/XSCALE* was used for the conventional method, the *CrystFEL* program suite was used for acoustic levitation, 4RLM^38^ and 4O34^39^, and *nXDS* was used for 4RLN^38^. It is clear in the figure that *CrystFEL* program suite has a tendency to give slightly higher B factors of the atoms, as compared with other data processing programs^38^,^39^,^40^.

The acquired datasets were first indexed with the *CrystFEL* program suite originally developed for SFX experiments^36^. The data processing allowed us to successfully index at least 25% of all the collected images. The number of images that we could successfully index was partly limited by the drift of the crystal away from the beam due to the orbiting of the crystal within the droplet. When the drift was small, a higher fraction of indexed images was obtained. For one of the datasets (see Table 1), 55% of the 3,600 images were successfully indexed. The indexed images were then integrated and merged. To solve the crystal structure, we applied the molecular replacement method for the datasets processed by the *CrystFEL* program suite. The detailed statistics are summarized in Table 1 for the solution of the structure from all of the indexed images (first column) and from the dataset with the high indexing rate (small dataset). The statistics were outstanding, and further structural refinement was successfully applied. Figures 3 and 4 summarize a part of the electron density and the temperature factor distribution of the residues.

**Figure 5:**
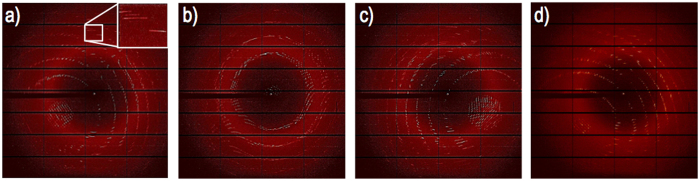
Snapshots of X-ray diffraction images from another dataset. These were captured at (**a**)11.39 s, (**b**) 11.44 s, (**c**) 11.49 s, and (**d**) 11.65 s (from left to right). The spinning of the crystal along an axis close to the z-axis (X-ray direction) is apparent from the symmetry of the diffraction spots and their rotation. The spin axis nearly coincided with the z-direction in the second image, whereas it was tilted from it by 4.2° in the last image. The spinning speed was ~1400° per second in this case as evaluated from the rotation of the diffraction spots. From the indexing of the diffraction spots, we found that the spinning axis approximately coincides with the c* direction.

From the analysis of crystal spinning orientation based on the movement of some of the diffraction spots, it appeared that the high (55%) indexing rate dataset was obtained when the spinning occurred along the vertical Y axis (see Fig. 1 for the definition of the axes), and the spinning rate was 0.2 to 1 rotation per second corresponding to a rotation angle of 0.5–2° per frame, which is comparable to the typical oscillation angle of 0.5° used in the conventional oscillation method. In contrast, when the crystal spun along the horizontal X or Z axes, the spinning speed appeared to be too fast (5–10° per frame), which resulted in streaked diffraction spots, as shown in Fig. 5 (see also supplementary movie 2), thereby making the data analysis difficult with the existing programs. Given the similar velocity between the crystallization liquid and the crystal studied here, it is likely that the internal circulation of the crystallization liquid has a similar rate. Thus, we estimated the maximum flow velocity near the surface of the droplet as ~10 mm/s, which is in good agreement with the reported values^32^.

### Reference X-ray diffraction experiment

To validate the refined molecular structure obtained above, we conducted a reference experiment with a crystal prepared in the same crystallization batch and used a conventional oscillation method at room temperature. We picked a large crystal (200 ×  500 ×  50 μ m^3^) and applied low X-ray dose to minimize the radiation damage. For this measurement, we acquired the diffraction images of up to 1.2 Å resolution. For comparison, we truncated the dataset to a 1.8 Å resolution limit. From a dataset with 900 diffraction images, we solved the crystal structure by using the molecular replacement method. The electron density and the temperature factor distribution of the solved structure are also shown for comparison in Figs 3 and 4.

## Discussion

The ability to solve the crystal structure from datasets collected by the acoustic levitation method shows that this is a promising method for fast-frame-rate data collection at room temperature. To clarify whether the structural integrity of the protein was affected by the ultrasonic acoustic pressure during the measurement, we analysed the solved and refined structure by comparing it with structures obtained under analogous conditions by the conventional oscillation method.

First, we compared the electron density of the solved crystal structures. Figure 3 shows solved crystal structures obtained with the acoustic levitation method (a) and the oscillation method (b). We found that the structures are nearly identical. In particular, the coordinate bonding of the water molecules (blue) and the metal atoms (red, sodium is right, cyan, chloride is left) is similar in both densities. These results are strong indications that the acoustic pressure has no effect on the molecular vibration and temperature. Second, we compared the differences in the disulphide bridge lengths that are normally used as a measure of the crystal damage caused by radiation^37^. As shown in Table 2, the disulphide bridge lengths of the solved and refined structures obtained from the both methods are identical within 0.02 Å. Notably, the effect of the acoustic pressure on the disulphide bridge length (normally used as a benchmark of the X-ray radiation damage) or on the crystal structure in general is not yet apparent. Nevertheless, the absence of an effect of the acoustic pressure on the disulphide bridge length is an indication that the lysozyme crystal structure remained intact during the levitation experiment.

Comparing the temperature factor distribution for individual residues provided another validation of the acoustic levitation method. Figure 4a,b show the temperature factor distributions of the solved structures obtained with the acoustic levitation method and the conventional method, with the same resolution limit. Although the distribution for the acoustic levitation method is higher by 13.9 Å^2^ than that for the conventional method, the overall B-factor is only 1.3 Å^2^ higher for the acoustic levitation method than for the conventional method (see Table 2). Additionally, the two distributions are highly similar (Fig. 4a,b). In synchrotron serial crystallography experiments on lysozyme, Botha *et al.*^38^ have carefully compared processing with two software packages *CrystFEL* and nXDS. They have shown that *CrystFEL* (the yellow line in Fig. 4c) systematically yields higher B-factors than nXDS (the green line in Fig. 4c), arguably because of the use of a global resolution cut-off^38^. Stellato *et al.*^39^ have found similarly high B-factors (the purple line in Fig. 4c) by using *CrystFEL* in capillary flow serial protein crystallography experiments. By processing our levitation method data with *CrystFEL* (the blue line in Fig. 4c), we found B-factors higher than the conventionally processed oscillation data (the red line in Fig. 4c). However, this difference was consistently well within the variation observed by Botha *et al.*^38^. Therefore, we believe that the higher temperature factor distribution for the acoustic levitation method was caused not by the crystal damage but by the CrystFEL program^38^,^39^,^40^.

Therefore it is likely that the high ultrasonic peak pressure in the acoustic droplet levitator had no apparent impact on the detailed coordination and folding of the protein structure, at least with lysozyme. We believe that, on the one hand this is because the actual acoustic pressure in the levitated droplet, which is held near the pressure node of the levitator, is low because of the low transmission of the acoustic radiation in the droplet. On the other hand the measurement time was short, with a 0.5- to 1.5 min exposure time and a 3- to 5 minutes levitation time at most.

It is also noteworthy that the relatively large amount of water and the kapton film in the acoustic levitation experiments did not affect the quality of the data in the highest resolution shell (1.8 Å), whereas the diffraction diffuse ring from water and kapton decreased the signal-to-noise ratio at an intermediate resolution (2.8–4.4 Å).

By using a detector with an even higher frame rate^4^, such as the EIGER 1 M (Dectris), which has a maximum frame rate of 3 kHz, it should be possible to collect the required dataset to solve a damage-free structures in a 1-second-timescale at room temperature in a synchrotron. Further, the analysis of a crystal spinning in a levitated droplet suggested that with a faster frame rate (and/or better control of the rotation speed), the streaking of the diffraction spots may be mitigated, and the index fraction of the images would be substantially improved. Therefore, future efforts will investigate the feasibility of the method studied here for proteins that are more fragile, such as smaller-sized lipidic cubic phase crystals, by using such kHz-frame-rate detectors.

For practical and challenging protein crystallography projects, data acquisition from microcrystals with the size of e.g. 20 μ m or smaller than used in this study will be essential. A key to successfully conducting such experiments is the delivery of single crystals in a smaller droplet, e.g. on the order of a few tens nanolitres or less, by a more sophisticated droplet injection system than that used here, which must overcome the adhesion of the crystallization buffer liquid to the capillary. Since a separate experiment demonstrated that a positional stability of the levitated droplets on the order of 10 μ m as realized in this study, it is likely that we can stably levitate droplets with a radius as small as a few hundred micrometres with the present acoustic levitator. When such a small droplet injection is realized, an X-ray diffraction experiment using crystals with a size of 20 μ m in levitated droplets will be feasible. It can be estimated that at a flux of 3 ×  10^12^ X-ray photons/s, the required number of diffraction images can be collected with a sufficient signal-to-noise ratio from crystals with a size of 20 μ m within ~1 s by using an available high repetition rate pixel detector with a frame rate of 3 kHz^4^, if the beam size is matched to the crystal size, the liquid volume in the drop and the air scattering before the crystal are minimized. The expected short data acquisition time of ~1 s should be able to minimize the radiation damage even at room temperature^4^,^41^.

In summary, we showed that it is possible to solve crystal structures from X-ray diffraction images obtained from single crystals in acoustically levitated droplets in air at room temperature. A combination of a pixel detector with a fast frame rate and a data analysis package developed for SFX allowed us to obtain a high resolution (1.8 Å) protein crystal structure with minimal sample consumption and high throughput. The statistics of the datasets were of outstanding quality, even in comparison to the conventional oscillation method. The electron density obtained was of high quality and had no apparent difference from that obtained in a conventional diffraction experiment. Although a high peak ultrasonic cavity pressure was used for the acoustic levitation of the droplet, the electron density, disulphide bridge lengths, and temperature factors indicated that the ultrasonic pressure caused no apparent crystal damage. We believe that the combination demonstrated here, i.e., a damage-free, high acquisition acoustic levitation, will produce a high efficiency pipeline of a fully automated crystal structure analysis from crystal harvesting to data acquisition. Such a system will greatly accelerate both the structural analysis of biomolecules and structure-based drug development.

## Methods

### Ultrasonic acoustic levitator

The acoustic levitator cavity consisted of a bolt-clamped Langevin-type lead-zirconate-titanate (PZT) ultrasound oscillator with a resonance frequency of ~38 kHz and a concave glass mirror reflector with a mirror radius of curvature equal to 40 mm. The transducer horn and the mirror diameter were equal to 20 mm. The transducer was driven by a variable frequency driver that maintained the transducer at resonance with a driving power of 2.0 W and a vibration amplitude as measured by Michelson interferometry equal to 3.2 μ m (rms), which corresponded to an rms velocity of 0.8 m/s and a pressure of 0.33 kPa (rms) in ambient air. The cavity pressure was monitored by a PZT transducer attached to the mirror reflector holder. As the gap is increased from zero, a series of resonances with a period of one half the wavelength of the acoustic radiation was observed. The liquid droplets can be levitated at the pressure nodes^6^. The peak cavity pressure was equal to ~3.6 kPa when the cavity was exactly at the third resonance. We loaded the droplets at a slightly reduced peak cavity pressure of ~2.1 kPa under the same transducer driving condition but at the gap distance 0.02 λ smaller than the third resonance gap, with an ultrasonic wavelength λ of 9 mm. We mounted the levitator on a separate translation stage so that we could align the position of the crystal in the levitating droplet to the X-ray beam independently of the adjustment of the levitation condition.

### X-ray diffraction experiment

Crystals of lysozyme were prepared by using the hanging-drop vapour diffusion method. The lysozyme protein solution (Alfa Aeser, Wardhill, MA, USA) had a concentration of 100 mg/ml in 50 mM Na acetate buffer at pH 4.5. The crystallization buffer consisted of 5% MPEG 5 K, 2 M NaCl, 50 mM Na acetate buffer at pH 4.5 and 25% ethylene glycerol. Then 10 μ l of the protein solution and the same amount of the crystallization buffer were mixed. The space group of crystals was *P*4_3_2_1_2, the unit cell parameters were *a* =  *b* =  80.02 Å, *c* =  37.69 Å, *α* =  *β* =  *γ* =  90.0°, for the conventional oscillation method, and *a* =  *b* =  80.23 Å, *c* =  38.50 Å, *α* =  *β* =  *γ* =  90.0°, for the acoustic levitation method. Droplets of approximately 4 μ l crystallization buffer, each containing a single lysozyme crystals of approximately 200 μ m size, were loaded in the middle node of the acoustic standing wave field. We first harvested the crystals from the crystallization plate with a nylon loop and then picked up the crystals with the buffer droplet held by a quartz capillary. We were able to detach the droplet from the capillary and levitate it by transporting it to the levitation point of the cavity. The peak cavity pressure was set to 2.1 ±  0.1 kPa (rms) when loaded. By inspecting the droplet with an in-line optical microscope, we confirmed that the residual drift of the droplet position was much less than the X-ray beam size of 40 ×  10 μ m^2^ (h ×  v, FWHM). From the optical inspection, we observed spinning of crystals (~1 rotation per second) as suggested by the internal recirculation of the levitated droplets^29^,^30^,^31^,^32^ and the orbiting of crystals (~1–2 Hz) within the droplets. During the experiments, the ambient temperature was 23 ±  0.5 °C.

### Data processing

The datasets collected with the EIGER X 16 M detector (Dectris, Baden, Switzerland) at 133 Hz in EIGER HDF5 format were first converted with in-house image converters into the *CrystFEL* HDF5 format with a calibrated mask image from Dectris for the detector. Next, they were indexed, integrated and merged in the *CrystFEL* 0.6.0 program suite^36^. We used *partialator* as the post-refinement method for the datasets. Because post refinement with ‘Gaussian’ or ‘sphere’ models yielded a large number of rejected diffraction spots, the ‘unity’ model was used to minimize the number of rejected spots. The statistics for the data processing and analysis are shown in Table 1. The final *Rwork/Rfree* values of the refined structures from the datasets recorded with the EIGER X 16 M and the EIGER X 16 M (small dataset) were 17.6/22.8 and 17.8/22.8, respectively. We removed water molecules, light metals and any other additional molecules from the model structure 4XJB. Molecular replacement was individually performed for each dataset with MOLREP. After rigid body refinement with REFMAC, we inspected into the Fo - Fc maps and assigned water molecules and light metals. In (Fig. 3a,b), 2Fo-Fc and Fo-Fc maps are superimposed for each dataset.

### Reference X-ray diffraction experiment

As a reference, a conventional X-ray diffraction experiment that used the standard oscillation method was conducted at room temperature with a crystal taken from the same crystallization batch as for the acoustic levitation experiments. After fishing a crystal on a kapton loop, we sealed it in a plastic capillary tube with a drop of crystallization buffer to avoid dehydration of the crystal during the experiment, using a room temperature experimental kit (MicroRT, MiTeGen, Ithaca, NY, USA). The crystal was rotated by a single phi axis goniometer at an oscillation range of 0.2°, and the diffraction images were collected at an exposure time of 0.1 second at a detector distance of 300 mm. We translated the crystal by 10 μ m for every 10 degrees of rotation to minimize the radiation damage. This procedure mimics the constant renewal of the crystal region exposed to X-rays in the levitation method, due to the spinning and orbiting of the crystal within the droplet. We collected 900 diffraction images in total. The dataset obtained was indexed and processed with *XDS* and *XSCALE*^42^. We then solved the crystal structure by applying the molecular replacement method using *MOLREP*^43^ and refined by using *REFMAC*^44^ in the *CCP4* program suite^45^. The PDB code 4XJB^46^ was used as the model structure for the molecular replacement. See Table 1 for the detailed processing statistics.

## Additional Information

**Data Availability**: Coordinates have been deposited in the Protein Data Bank under accession codes 5FDJ (levitation all), 5FEK (levitation small dataset) and 5FEL (conventional method).

**How to cite this article**: Tsujino, S. and Tomizaki, T. Ultrasonic acoustic levitation for fast frame rate X-ray protein crystallography at room temperature. *Sci. Rep.* **6**, 25558; doi: 10.1038/srep25558 (2016).

## Supplementary Material

Supplementary Information

Supplementary Movie 1

Supplementary Movie 2

## Figures and Tables

**Table 1:** Experimental conditions and statistics of the data processing.

Method	Acoustic levitation	Acoustic levitation (small dataset)	Oscillation
Detector	EIGER X 16 M	EIGER X 16 M	PILATUS 6 M
Data processing suite	*CrystFEL 0.6.0*	*CrystFEL 0.6.0*	*XDS/XSCALE*
Wavelength (Å)	1.000	1.000	1.000
Flux (photons/s)	1.0 × 10^12^	1.0 × 10^12^	8.0 × 10^9^
Dose per frame (Gy)	2430	2430	647
Frame rate (Hz)	133	133	10
Oscillation range (°)	–	–	0.2
Space group	P4_3_2_1_2	P4_3_2_1_2	P4_3_2_1_2
Unit cell *a*/*c* (Å)	80.23/38.50	80.23/38.50	80.02/37.69
Total no. of images	36,000	3,600	900
No. of indexed images	7,283	2,045	900
Resolution range (Å)	50.0–1.8 (1.83–1.80)	50.0–1.8 (1.83–1.80)	50.0–1.8 (1.91–1.80)
Total no. of spots	4,719,209 (136,313)	1,422,292 (20,757)	150,034 (23,866)
Redundancy	406.7 (121.1)	114.7 (29.1)	6.96 (6.90)
Completeness (%)	99.7 (97.4)	99.0 (58.7)	99.9 (99.9)
I/sigma	15.90 (10.11)	11.62 (7.23)	27.89 (13.55)
Rmerge	–	–	5.0 (11.4)
Rsplit	7.46 (13.74)	9.96 (18.77)	–
cc1/2	0.98 (0.81)	0.972 (0.71)	0.999 (0.99)

**Table 2:** Statistics of the data analysis and structure refinement of the lysozyme crystals.

Method	Acoustic levitation	Acoustic levitation (small dataset)	Oscillation
Detector	EIGER X 16 M	EIGER X 16 M	PILATUS 6 M
Contrast from *MOLREP*	10.83	15.44	17.76
R*work* (%)	17.6	17.8	15.0
R*free* (%)	22.8	22.8	19.9
Overall B-factor (Å^2^)	45.0	43.7	43.8
S_6_ – S_127_ (Å)	1.98	1.99	1.96
S_30_ – S_115_ (Å)	2.03	2.03	2.03
S_64_ – S_80_ (Å)	2.01	2.01	2.04
S_76_ – S_94_ (Å)	2.03	2.03	2.06

## References

[b1] Henrich B. *et al.* PILATUS: A single photon counting pixel detector for X-ray applications. Nuclear Instruments and Methods in Physical Research A 607, 247 (2009).

[b2] Selmer M. *et al.* Structure of the 70S ribosome complexed with mRNA and tRNA. Science 313, 1935–1942 (2006). 16959973 10.1126/science.1131127

[b3] Owen R. L. Outrunning free radicals in room-temperature macromolecular crystallography. Acta Cryst. D68, 810 (2012). 10.1107/S0907444912012553 PMC4791751 22751666

[b4] Dinapoli R. *et al.* EIGER: Next generation single photon counting detector for X-ray applications. Nuclear Instruments and Methods in Physics Research A 650, 79 (2011).

[b5] Fraser J. S. *et al.* Accessing protein conformational ensembles using room-temperature X-ray crystallography. PNAS 108, 16247 (2011). 21918110 10.1073/pnas.1111325108 PMC3182744

[b6] Keedy D. A. *et al.* Mapping the conformational landscape of a dynamic enzyme by multitemperature and XFEL crystallography. eLife (2015), doi: 10.7554/eLife.07574. PMC4721965 26422513

[b7] King L. V. On the Acoustic Radiation Pressure on Spheres. Proc. R. Soc. Lond. A 147, 212 (1934).

[b8] Tanaka H., Wada Y., Mizuno Y. & Nakamura K. Behavior of ultrasonically levitated object above reflector hole. Jap. J. Appl. Phys. 52, 100201 (2013).

[b9] Foresti D., Nabavi M., Klingauf M., Ferrari A. & Poulikakos D. Acoustophoretic contactless transport and handling of matter in air. PNAS 110, 12549 (2013). 23858454 10.1073/pnas.1301860110 PMC3732964

[b10] Ochiai Y., Hoshi T. & Rekimoto J. Three-Dimensional Mid-Air Acoustic Manipulation by Ultrasonic Phased Arrays. PLos ONE 9, e97590 (2014). 24849371 10.1371/journal.pone.0097590 PMC4029622

[b11] Xie W. J., Cao C. D., Lu Y. J. & Wei B. Levitation of Iridium and Liquid Mercury by Ultrasound, Phys. Rev. Lett. 89, 104304 (2002). 12225198 10.1103/PhysRevLett.89.104304

[b12] Xie W. J., Cao C. D., Lü Y. J., Hong Z. Y. & Wei B., Acoustic method for levitation of small living animals. Appl. Phys. Lett. 89, 214102 (2006).

[b13] Sundvik M., Nieminen H. J., Salmi A., Panula P. & Hæggström E. Effects of acoustic levitation on the development of zebrafish, Danio rerio, embryos. Scientific Reports 5, 13596 (2015). 26337364 10.1038/srep13596 PMC4559763

[b14] Yarin A., Brenn G., Kastner O., Rensink D. & Tropea C. Evaporation of acoustically levitated droplets, Journal of Fluid Mechanics 399, 151 (1999).

[b15] Lü Y. J., Xie W. J. & Wei B., Observation of ice nucleation in acoustically levitated water drops. Applied Physics Letters 87, 184107 (2005).

[b16] Wulsten E. & Lee G. Surface temperature of acoustically levitated water microdroplets measured using infra-red thermography. Chemical Engineering Science 63, 5420 (2008).

[b17] Leiterer J., Delißen F., Emmerling F., Thünemann A. F. & Panne U. Structure analysis using acoustically levitated droplets. Anal Bioanal Chem 391, 1221(2008). 18373085 10.1007/s00216-008-2011-2

[b18] Delißen F., Leiterer J., Bienert R., Emmerling F. & Thünemann A. F. Agglomeration of proteins in acoustically levitated droplets. Anal Bioanal Chem 392, 161 (2008). 18607573 10.1007/s00216-008-2252-0

[b19] Geng D. L., Xie W. J. & Wie B. Containerless solidification of acoustically levitated Ni–Sn eutectic alloy. Appl Phys A 109, 239 (2012).

[b20] Santesson S. & Nilsson S. Airborne chemistry: acoustic levitation in chemical analysis. Anal Bioanal Chem 378, 1704 (2004). 14762640 10.1007/s00216-003-2403-2

[b21] Tuckermann R., Puskar L., Zavabeti M., Sekine R. & McNaughton D. Chemical analysis of acoustically levitated drops by Raman spectroscopy, Anal Bioanal Chem 394, 1433 (2009). 19418043 10.1007/s00216-009-2800-2 PMC3085753

[b22] Pierre Z. N., Field C. R. & Scheeline A. Sample Handling and Chemical Kinetics in an Acoustically Levitated Drop Microreactor. Anal. Chem. 81, 8496 (2009). 19769373 10.1021/ac901400y PMC2761965

[b23] Weber R. J. K. *et al.* Acoustic levitation: recent developments and emerging opportunities in biomaterials research, Eur Biophys J. 41, 397 (2012). 22038123 10.1007/s00249-011-0767-3

[b24] Scheeline A. & Behrens R. L. Potential of levitated drops to serve as microreactors for biophysical measurements. Biophysical Chemistry 165, 1 (2012). 22498502 10.1016/j.bpc.2012.03.008

[b25] Soares A. S. *et al.* Acoustically Mounted Microcrystals Yield High Resolution X-ray Structures. Biochemistry 50, 4399 (2011). 21542590 10.1021/bi200549x PMC3144476

[b26] Roessler C. G. *et al.* Acoustic methods for high-throughput protein crystal mounting at next-generation macromolecular crystallographic beamlines. J. Synchrotron Rad. 20, 805 (2013). 10.1107/S0909049513020372 PMC3747951 23955046

[b27] Santesson S. *et al.* Screening of Nucleation Conditions Using Levitated Drops for Protein Crystallization. Anal. Chem. 75, 1733–1740 (2003). 12705610 10.1021/ac020496y

[b28] Yin X. *et al.* Hitting the target: fragment screening with acoustic *in situ* co-crystallization of proteins plus fragment libraries on pin-mounted data-collection micromeshes. Acta Cryst D 70, 1177 (2014). 24816088 10.1107/S1399004713034603 PMC4014116

[b29] Trinh E. H. & Robey J. L. Experimental study of streaming flows associated with ultrasonic levitators. Physics of Fluids 6, 3567 (1994).

[b30] Zhao H., Sadhal S. S. & Trinh E. H. Internal circulation in a drop in an acoustic field. J. Acoust. Soc. Am. 106, 3289 (1999). 10615685 10.1121/1.428182

[b31] Abe Y., Hyuga D., Yamada S. & Aoki K. Study on Internal Flow and Surface Deformation of Large Droplet Levitated by Ultrasonic Wave. Annals of the New York Academy of Sciences, Interdisciplinary Transport Phenomena in The Space Sciences 1077, 49 (2006). 10.1196/annals.1362.050 17124114

[b32] Ishii H., Hasegawa K., Kaneko A. & Abe Y. Internal and external flow structure and mass transport phenomena of an acoustically levitated droplet.Transactions of the Jpn. Soc. Mech. Eng. 794, 1696 (2012).

[b33] Bingel-Erlenmeyer R. *et al.* SLS Crystallization Platform at Beamline X06DA—A Fully Automated Pipeline Enabling *in situ* X-ray Diffraction Screening. Cryst. Growth Des. 11, 916 (2011).

[b34] Axford D. *et al.* *In situ* macromolecular crystallography using microbeams. Acta Cryst. D68, 592 (2012). 10.1107/S0907444912006749 PMC4791750 22525757

[b35] Cipriani F. *et al.* CrystalDirect: a new method for automated crystal harvesting based on laser-induced photoablation of thin films. Acta Crystallogr D Biol Crystallogr. 68, 1393 (2012). 22993093 10.1107/S0907444912031459

[b36] White T. A. *et al.* CrystFEL: a software suite for snapshot serial crystallography. J. Appl. Cryst. 45, 335–341 (2012).

[b37] Burmeister W. P. Structural changes in a cryo-cooled protein crystal owing to radiation damage. Acta Cryst. D56, 328 (2000). 10.1107/s0907444999016261 10713520

[b38] Botha S. *et al.* Room-temperature serial crystallography at synchrotron X-ray sources using slowly flowing free-standing high-viscosity microstreams. Acta Cryst. D71, 387 (2015). 10.1107/S1399004714026327 25664750

[b39] Stellato F. *et al.* Room-temperature macromolecular serial crystallography using synchrotron radiation. IUCrJ 1, 204–212 (2014). 10.1107/S2052252514010070 PMC4107920 25075341

[b40] Sawaya M. R. *et al.* Protein crystal structure obtained at 2.9 Å resolution from injecting bacterial cells into an X-ray free-electron laser beam. PNAS 111, 12769 (2014). 25136092 10.1073/pnas.1413456111 PMC4156696

[b41] Warkentin M. *et al.* Global radiation damage at 300 and 260 K with dose rates approaching 1 MGy s-1. Acta Cryst. D68, 124 (2012). 10.1107/S0907444911052085 PMC3266852 22281741

[b42] Kabsch W. XDS. Acta Cryst. D66, 125–132 (2010). 10.1107/S0907444909047337 PMC2815665 20124692

[b43] Vagin A. & Teplyakov A. MOLREP: an automated program for molecular replacement. J. Appl. Cryst. 30, 1022–1025 (1997).

[b44] Murshudov G. N., Vagin A. A. & Dodson E. J. Refinement of Macromolecular Structures by the Maximum-Likelihood Method. Acta Crystallogr. D53, 240–255 (1997). 10.1107/S0907444996012255 15299926

[b45] Winn M. D. *et al.* Overview of the CCP4 suite and current developments. Acta Cryst. D67, 235–242 (2011). 10.1107/S0907444910045749 PMC3069738 21460441

[b46] Huang C.-Y. *et al.* In meso *in situ* serial X-ray crystallography of soluble and membrane proteins. Acta Cryst. D71, 1238 (2015). 10.1107/S1399004715005210 PMC4461204 26057665

[b47] McNicholas S., Potterton E., Wilson K. S. & Noble M. E. M. Presenting your structures: the CCP4mg molecular-graphics software. Acta Cryst. D67, 386 (2011). 10.1107/S0907444911007281 PMC3069754 21460457

