# Supplementary material for: Ultrasonic acoustic levitation for fast frame rate X-ray protein crystallography at room temperature
Source: Sci Rep. 2016 May 6;6:25558. doi: 10.1038/srep25558 (PMC4858681; doi:10.1038/srep25558)
Supplement: Supplementary Information [file srep25558-s1.pdf]

Ultrasonic acoustic levitation for fast frame rate X-ray protein crystallography at room temperature

Soichiro Tsujino and Takashi Tomizaki

Supplementary information file

Supplementary video 1: X-ray diffraction images of single lysozyme crystal between 10.5 s and 10.9 s (after the start of data acquisition), displaying the spinning of the crystal with a small precession of c* direction. The image was captured by 133 Hz pixel detector, cf. Fig. 2.

Supplementary video 2: X-ray diffraction images of single lysozyme crystal (between 10.9-10.7 s after the start of data acquisition). The crystal spun along an axis close to the z-axis (X-ray direction), however the spinning speed of ~1400{degree sign} per second is apparent from the symmetry of the diffraction spots and their rotation resulted in streaked diffraction spots, cf. Fig. 3.
